# Hippocampal region metabolites and cognitive impairment in patients with general paresis: based on ^1^H-proton magnetic resonance spectroscopy

**DOI:** 10.3389/fphar.2024.1382381

**Published:** 2024-04-17

**Authors:** Xin Che, Tianyang Miao, Haishan Shi, Zezhi Li, Yuping Ning

**Affiliations:** ^1^ The Affiliated Brain Hospital, Guangzhou Medical University, Guangzhou, China; ^2^ Guangdong Engineering Technology Research Center for Translational Medicine of Mental Disorders, Guangzhou, China; ^3^ Key Laboratory of Neurogenetics and Channelopathies of Guangdong Province and the Ministry of Education of China, Guangzhou Medical University, Guangzhou, China

**Keywords:** general paresis, general paresis of insane, magnetic resonance spectroscopy, *Treponema pallidum*, hippocampus

## Abstract

**Background:**

This study utilizes Hydrogen proton magnetic resonance spectroscopy (^1^H-MRS) to investigate metabolite concentrations in the bilateral hippocampus of general paresis (GP) patients.

**Methods:**

A total of 80 GP patients and 57 normal controls (NCs) were enrolled. Metabolite ratios in the bilateral hippocampus were measured using ^1^H-MRS. Cognitive function was assessed using the Mini-Mental State Examination (MMSE). Based on MMSE scores, participants were categorized into normal control, mild cognitive impairment, and moderate-severe dementia groups. Metabolite ratios (N-acetylaspartate (NAA)/creatine (Cr), choline (Cho)/creatine (Cr), N-acetylaspartate (NAA)/choline (Cho), myoinositol (MI)/creatine (Cr), choline (Cho)/N-acetylaspartate (NAA)) were compared between groups, and correlations between metabolite ratios and cognitive performance were examined.

**Results:**

MMSE scores progressively decreased in the normal, mild cognitive impairment, and moderate-severe dementia groups (*p* < 0.001). The moderate-severe dementia group showed significantly lower NAA/Cr ratios in the left hippocampus region (L-NAA/Cr ratios) (*p* < 0.001) and higher Cho/NAA ratios in the left hippocampus region (L-Cho/NAA ratios) (*p* < 0.05) compared to the other groups. However, differences in L-NAA/Cr and L-Cho/NAA ratios between the mild cognitive impairment group and the NC group were not significant in the hippocampus region (*p* > 0.05). NAA/Cho and NAA/Cr ratios in the right hippocampus region (R-NAA/Cho and R-NAA/Cr ratios) in the moderate-severe dementia group were lower than those in the control group (*p* < 0.05). No correlation was found between metabolite ratios and MMSE scores in bilateral hippocampus regions.

**Conclusion:**

There are distinctive metabolic characteristics in the hippocampus of GP patients. GP patients exhibited lower NAA/Cr and NAA/Cho ratios in the bilateral hippocampus, indicating neuron loss in these areas, which may become more pronounced as the disease progresses.

## Introduction

Although syphilis, an infection caused by *Treponema pallidum* (*T. pallidum*) primarily transmitted through sexual behaviors, was nearly eradicated from China 50 years ago, it has experienced a gradual resurgence (Wang et al., 2011). Furthermore, reports indicate that the incidence of syphilis continued to rise between January 2005 and December 2020 in China (Zheng et al., 2023). Researchers have observed the presence of *T. pallidum* and an increased number of spirochetal plaques in the cerebral cortex of certain patients diagnosed with general paresis (GP), suggesting the invasion of the central nervous system by *T. pallidum* in individuals affected by syphilis. When the cerebral cortex shows extensive deterioration or manifests dementia symptoms, this condition is commonly referred to as “general paralysis of the insane” (GPI), “dementia paralytica,” or simply “general paralysis” (Miklossy, 2015; Chakravarthi and Joshi, 2021; Ha et al., 2024).

Hydrogen proton magnetic resonance spectroscopy (^1^H-MRS) is a non-invasive technique employed to explore alterations in neurogenesis-related metabolites within the live human hippocampus concerning particular tasks or disease states. This method allows for the assessment of energy metabolism, neuronal function, and glial response in the human brain, particularly useful in the study of neurodegenerative disorders (Maul et al., 2020), brain tumors (Tomiyasu and Harada, 2022), central nervous system infection (Liu et al., 2023) and psychiatric disorders (Port, 2020). N-acetylaspartate (NAA), choline (Cho), myoinositol (MI), and creatine (Cr) are frequently identified metabolites in ^1^H-MRS. NAA is indicative of neuron densities, Cho is linked to membrane breakdown and turnover, MI serves as an organic osmolyte and a marker for glial cells, while Cr is recognized as a marker for energy metabolism (Lucassen et al., 2020; Tomiyasu and Harada, 2022).

Although significant abnormal structural changes may not be apparent in the early stages of Alzheimer’s Disease (AD), in HIV-positive individuals, and in patients with neurosyphilis, metabolic disorders are detected through ^1^H-MRS abnormalities. These abnormalities unveil distinct metabolic patterns across various brain regions, which are associated with the progression of the respective diseases (Pw et al., 2014; Zeng et al., 2021).

It has been established that patients with AD exhibit deposition of Aβ and phosphorylated-tau in the brain. Similarly, patients with GP demonstrate pathophysiological features similar to those observed in AD (Danics et al., 2021). Morphologically, the distribution of spirochete colonies in patients with GP mirrors the deposition pattern of senile plaques. Spirochetes are predominantly found in the hippocampus and frontal cortex, and their buildup correlates with consequential alterations. GP patients commonly experience a broad decline in cognitive function, chiefly marked by memory impairment, frequently accompanied by mental symptoms like impulsivity and delusions (Chiara et al., 2023). These symptoms could indicate neuronal damage in regions such as the hippocampus, and ^1^H-MRS may indirectly indicate the extent of such damage. However, there has been limited exploration of metabolite concentrations in the hippocampus of patients with GP using ^1^H-MRS, and even fewer studies have investigated the correlations between metabolite levels and cognitive function in these patients.

Therefore, through the utilization of ^1^H-MRS, we assessed the metabolic alterations in the hippocampus of patients with GP and examined the associations between metabolite levels in the bilateral hippocampus and the cognitive function of GP patients. This investigation aimed to reflect the extent of neuronal damage and the severity of the disease.

## Subjects and methods

### Subjects

All patients were recruited from the inpatient units of the Affiliated Brain Hospital of Guangzhou Medical University, and written informed consent was obtained from patients or their guardians, particularly in cases of severe cognitive impairment.

Comprehensive assessments were conducted, involving thorough history taking, physical and neurological examinations, neuropsychological assessments, appropriate laboratory tests, and magnetic resonance imaging (MRI) scans. Special attention was given to laboratory tests, specifically checking vitamin B12, folate, and thyroid hormone status to rule out dementia caused by anemia or thyroid hypofunction, thereby enhancing the accuracy of clinical diagnoses.

Cognitive function was evaluated using the Mini-Mental State Examination (MMSE), and diagnoses were reviewed by a senior neurologist with subspecialty training in neurodegenerative disorders.

Patients meeting the criteria for general paresis were carefully selected based on the following criteria: 1) meeting the DSM-IV criteria for dementia with a duration exceeding 1 year; 2) testing positive for the rapid plasma reagin (RPR) or the toluidine red unheated serum test (TRUST), and *T. pallidum* hemagglutination (TPHA) in both serum and cerebrospinal fluid (CSF); 3) having white cell counts in CSF of 10 × 10^6^/L and/or protein levels exceeding 500 mg/L. Exclusion criteria included patients with concurrent human immunodeficiency virus infection or cerebral infection caused by other microorganisms (Wang et al., 2011; Haishan et al., 2015).

The criteria for the normal control group included: 1) absence of neurological or psychiatric disorders; 2) no abnormal findings in conventional brain MR imaging; 3) no complaints of cognitive disorders; 4) an MMSE total score of 27 or higher.

### MRI data acquisition

**FIGURE 1:**
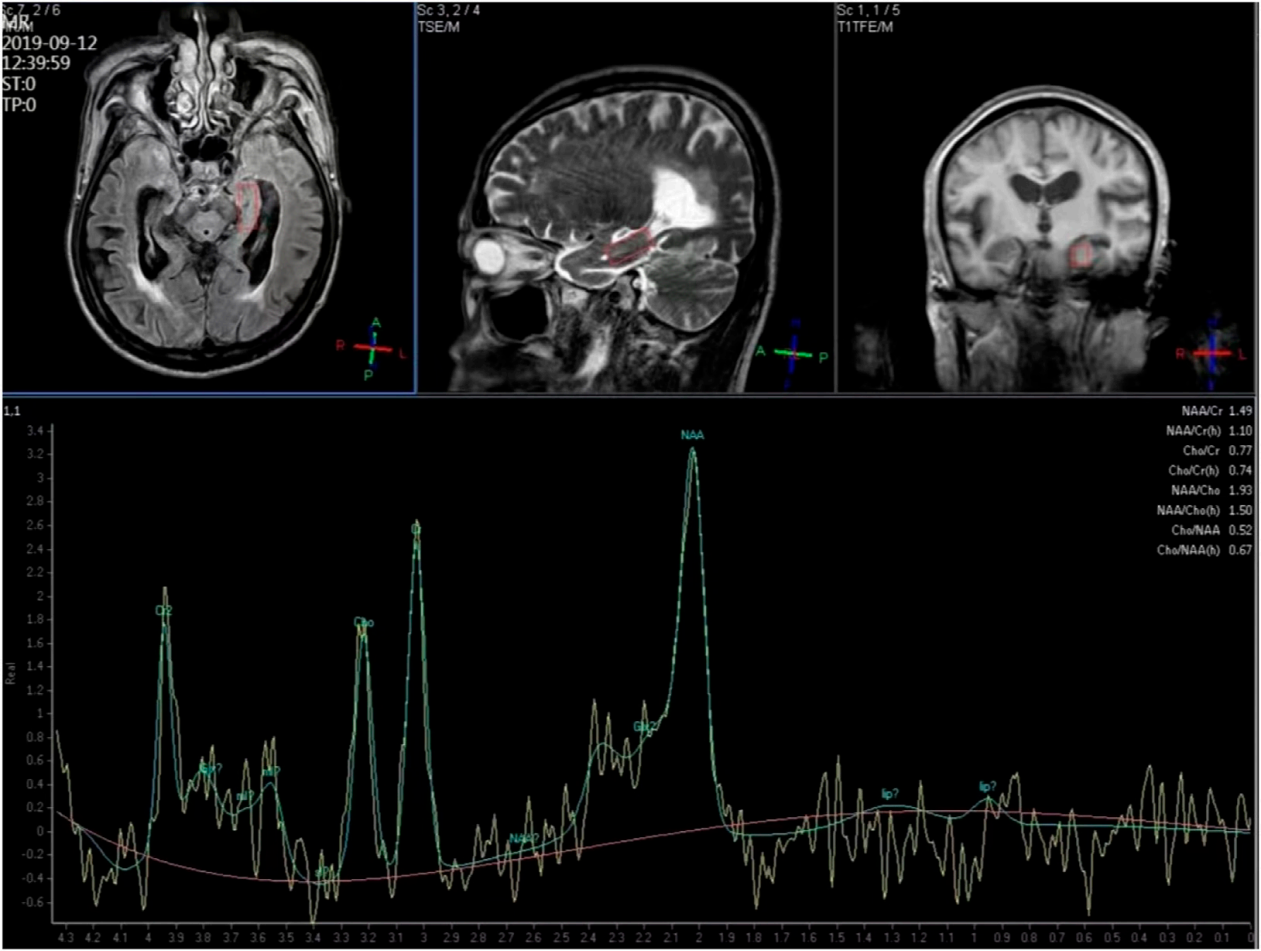
Hydrogen proton magnetic resonance spectroscopy for the left hippocampus region.

^1^H-MRS and structural MRI examinations were conducted using a Philips 3T Achieva MR scanner (Philips Medical Systems, Best, Netherlands). We selected the hippocampus as the region of interest. T1-weighted images facilitated tissue segmentation, and manual editing of the hippocampus was performed. A point-resolved spectroscopy (PRESS) sequence was employed to simultaneously acquire water-suppressed ^1^H-MRS from the bilateral hippocampus regions. The timing parameters were set at TR/TE/NSA 2000ms/35ms/128, with an in-plane resolution of 25 × 10 mm^2^ and a 10-mm-thick section, aligned approximately along the long axis of the hippocampus (Figure 1).

Structural MRI together with ^1^H-MRS, including shimming and adjustments, required approximately 40 min of acquisition time. Peak areas of NAA, Cr, Cho and MI were estimated using fully automated spectral-fitting software. The ratios of the area under each peak were expressed relative to Cr in each spectrum. Voxel placements for spectroscopy and all data analysis were carried out by a trained radiologist who was blind to each subject’s diagnosis.

### Statistical analysis

G*power software was used to calculate the power and effect size. A total of at least 52 subjects were included, with a minimum of 26 subjects in each group. Data analysis was conducted using the Statistical Package for Social Sciences (SPSS, version 29). Descriptive statistics including mean ± standard deviation (M±SD), median, and percentiles were calculated. Demographic and clinical variables between two groups were assessed using chi-square analysis and Independent-Sample *t*-Test. Two-group comparisons were performed with Independent-Sample *t*-Test or Mann-Whitney U Test. For comparisons among three groups, Oneway-ANOVA or Kruskal-Wallis H Test was employed, followed by *post hoc* Bonferroni’s test for multiple comparisons. This analysis included demographic and clinical characteristics data, energy metabolism data, and cognitive assessments.

Correlations between ^1^H-MRS data and cognitive scale scores were examined using Spearmen correlations, and false discovery rate (FDR) correction was applied. A *p*-value less than 0.05 was considered statistically significant, and all tests were two-tailed.

## Results

### Demographic and clinical charateristics

**FIGURE 2:**
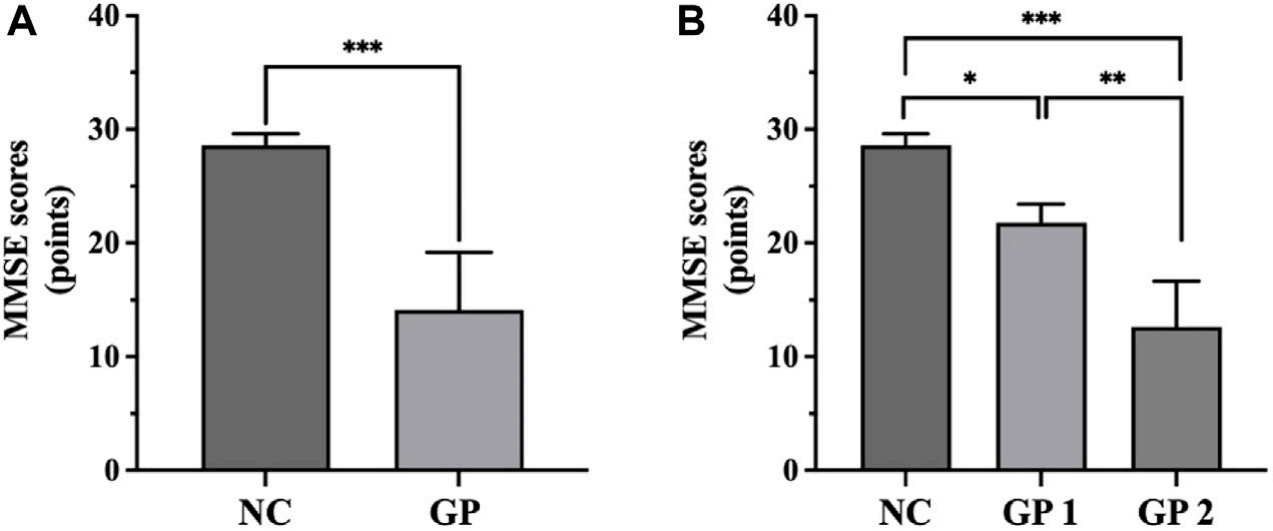
Comparisons of MMSE scores among groups. **(A)** GP group had significant lower scores on MMSE test. **(B)** GP 2 group had significant lower scores on MMSE test compared to the NC and GP 1 groups. NC: control group. GP 1: mild cognitive impairment group. GP 2: moderate-severe dementia group. ∗*p* < 0.05, ∗∗*p* < 0.01, ∗∗∗*p* < 0.001.

We recruited 80 patients with GP and 57 NC subjects. There were no significant differences in age between the GP and the NC groups (*p* > 0.05). GP group had significant lower scores on MMSE test (*p* < 0.001) (Figure 2). There were significant differences between groups. Participants in each group were categorized based on their level of education. No significant differences were observed between the two groups in terms of education level (Table 1).

**TABLE 1:** Demographic and clinical characteristics of GP and NC subjects.

	NC	GP	t/H/*x* ^*2*^	*p*	*Post Hoc*
n	57	80	—	—	—
Male/Female	24/33	68/12	27.77	<0.001	
Age (years)	58.40 ± 7.41	55.76 ± 9.15	1.80	0.07	
MMSE (points)	29.00 (28.00, 29.00)	14.00 (11.25, 18.00)	−9.99	<0.001	NC >GP
Education level (illiterate/elementary education/middle school education/bachelor’s degree and above)	1/20/30/6	1/24/44/11	0.91^a^	0.88	

^a^ Fisher’s exact test.

### Metabolite concentrations in the GP and NC groups

**FIGURE 3:**
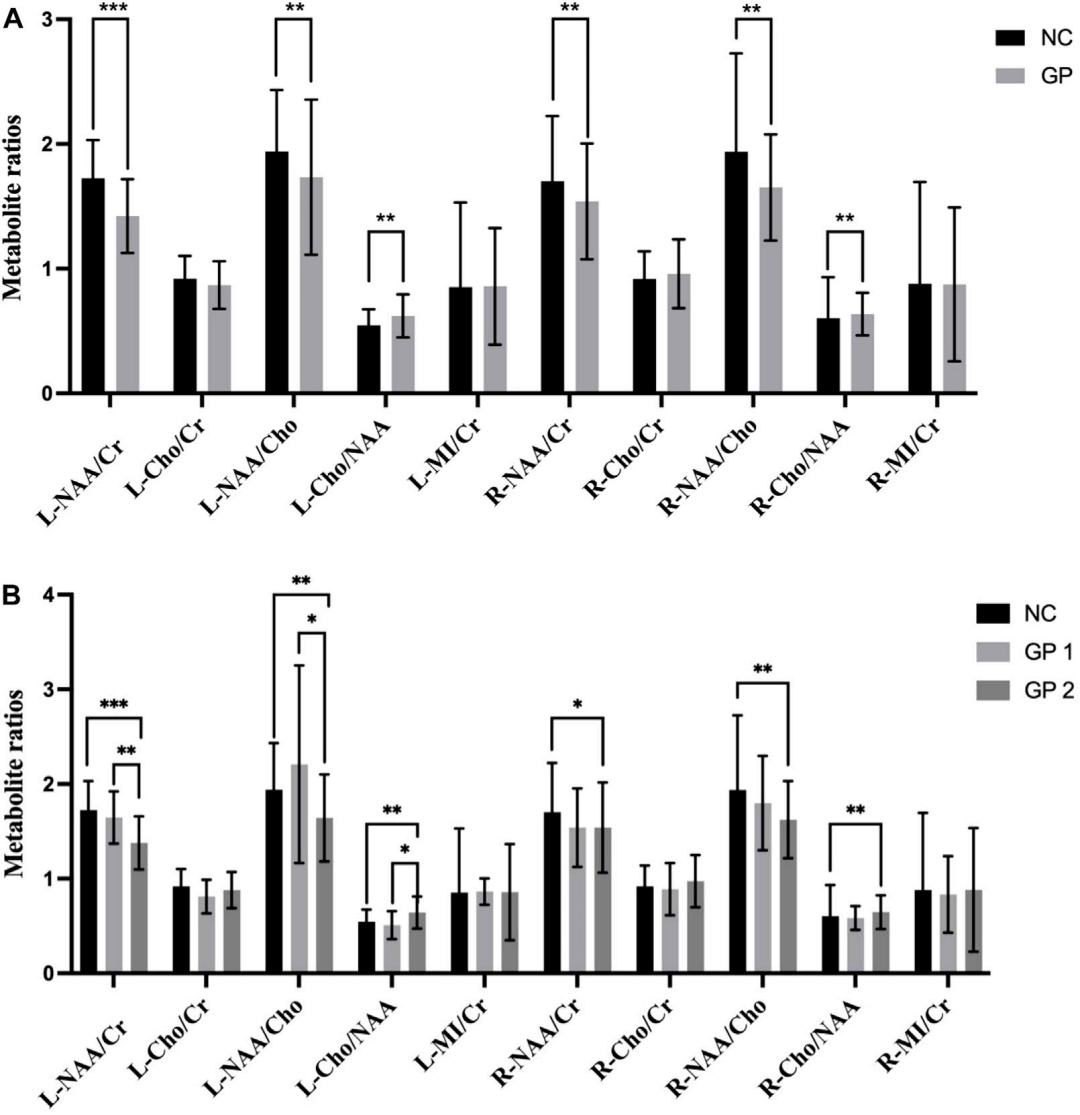
Comparisons of the ratios of metabolite concentrations in bilateral hippocampus. **(A)** Metabolite concentrations of NC and GP groups were compared. **(B)** Metabolite concentrations in bilateral hippocampus of NC, GP 1 group and GP 2 group were compared. L: the left hippocampus region. R: the right hippocampus region. NAA: N-acetylaspartate. Cr: creatine. Cho: choline. MI: myoinositol. ∗*p* < 0.05, ∗∗*p* < 0.01, ∗∗∗*p* < 0.001.

Metabolite concentrations in bilateral hippocampus of the GP and the NC groups were compared. GP patients showed significantly lower NAA/Cr and NAA/Cho ratios in bilateral hippocampus region (*p* < 0.05) compared to the NC subjects. It also exhibited a significantly higher Cho/NAA ratios in bilateral hippocampus region (*p* < 0.05) compared to the NC subjects. Differences in Cho/Cr and MI/Cr ratios between the GP and the NC groups were not significant in bilateral hippocampus region (*p* > 0.05) (Table 2; Figure 3).

**TABLE 2:** Comparisons of metabolite concentrations of GP and NC subjects.

	NC	GP	t/H	*p*	*Post Hoc*
L-NAA/Cr	1.72 ± 0.31	1.42 ± 0.30	5.81	<0.001	NC >GP
L-Cho/Cr	0.92 ± 0.18	0.87 ± 0.19	1.56	0.12	—
L-NAA/Cho	1.93 (1.53, 2.17)	1.65 (1.37, 1.88)	−2.99	0.003	NC >GP
L-Cho/NAA	0.52 (0.46, 0.65)	0.61 (0.53, 0.73)	−2.79	0.005	NC <GP
L-MI/Cr	0.71 (0.53, 0.92)	0.77 (0.60, 0.96)	−1.39	0.17	—
R-NAA/Cr	1.64 (1.45, 1.87)	1.49 (1.27, 1.71)	−2.67	0.008	NC >GP
R-Cho/Cr	0.90 (0.77, 1.08)	0.90 (0.77, 1.08)	−0.28	0.78	—
R-NAA/Cho	1.87 (1.49, 2.24)	1.59 (1.36, 1.85)	−3.10	0.002	NC >GP
R-Cho/NAA	0.54 (0.45, 0.67)	0.63 (0.53, 0.73)	−2.97	0.003	NC <GP
R-MI/Cr	0.70 (0.35, 1.00)	0.81 (0.52, 1.07)	−1.11	0.27	—

According to the MMSE scores, participants were recruited and divided into three groups: 57 normal controls with MMSE score 27 to 30 (control group, NC group), 13 patients with MMSE score 20 to 26 (mild cognitive impairment group, GP 1 group) and 67 patients with MMSE score 0 to 19 (moderate-severe dementia group, GP 2 group).

There were no significant differences in age and education level between three groups (*p >* 0.05). Moderate-severe dementia group had significant lower scores on MMSE test compared to the others (*p* < 0.05). There were differences in sex between three groups (Table 3; Figure 2).

**TABLE 3:** Demographic and clinical characteristics among the group.

	NC	GP 1	GP 2	t/H/*x* ^*2*^	*p*
n	57	13	67	—	—
Male/Female	24/33	12/1	56/11	28.14	<0.001
Age (years)	58.40 ± 7.41	54.54 ± 8.69	56.00 ± 9.28	1.77	0.17
MMSE (points)	29.00 (28.00, 29.00)	22.00 (20.00, 23.00)	13.00 (10.00, 15.00)	110.94	<0.001
Education level (illiterate/elementary education/middle school education/bachelor’s degree and above)	1/20/30/6	0/5/5/3	1/19/39/8	3.75^a^	0.75

^a^ Fisher’s exact test.

### Metabolite concentrations in the mild cognitive impairment group, moderate-severe dementia group and NC groups

Metabolite concentrations in bilateral hippocampus of three groups were compared. GP patients showed significantly lower NAA/Cr and NAA/Cho ratios in the left hippocampus region (L-NAA/Cr and L-NAA/Cho ratios) compared to the NC subjects. So we did ANOVA test for multiple comparisons. Moderate-severe dementia groups showed significantly lower L-NAA/Cr and L-NAA/Cho ratios (*p* < 0.05), compared with the other two groups. But differences in L-NAA/Cr and L-NAA/Cho ratios between the mild cognitive impairment group and the NC groups were not significant (*p* > 0.05).

It also exhibited a significantly lower NAA/Cho and NAA/Cr ratios in right side of the hippocampus region (R-NAA/Cho and R-NAA/Cr ratios) in moderate-severe group (*p* < 0.05) compared to the NC subjects. Additionally, there were no difference between the GP 2 and GP 1 groups, nor between the GP 1 and NC groups (*p* > 0.05).

Conversely, Moderate-severe dementia group showed significantly higher L-Cho/NAA ratios (*p* < 0.001), compared with the other two groups. But differences in L-Cho/NAA ratios between the GP 1 and NC group were not significant (*p* > 0.05). It also showed a significantly higher Cho/NAA ratios in right side of the hippocampus region (R-Cho/NAA ratios) in GP 2 group (*p* < 0.05) compared to the NC subjects. We found no significant difference in R-Cho/NAA between the GP 1 and NC groups. Furthermore, there was no significant difference in R-Cho/NAA levels between the GP 1 and GP 2 groups (*p* > 0.05).

When we compared the differences of Cho/Cr and MI/Cr ratio in bilateral hippocampus region observed in all groups by means of Kruskal-Wallis Test analyze we did not find significant differences (*p* > 0.05) (Table 4; Figure 3).

**TABLE 4:** Comparisons of metabolite concentrations among the groups.

	NC	GP 1	GP 2	*F/H*	*p*	Two-by-two comparison
L-NAA/Cr	1.72 ± 0.31	1.65 ± 0.28	1.38 ± 0.28	22.52	<0.001	NC, GP 1> GP 2
L-Cho/Cr	0.92 ± 0.18	0.81 ± 0.18	0.88 ± 0.19	1.95	0.15	—
L-NAA/Cho	1.93 (1.53, 2.17)	1.95 (1.69, 2.20)	1.60 (1.30, 1.82)	17.31	<0.001	NC, GP 1 >GP 2
L-Cho/NAA	0.52 (0.46, 0.65)	0.51 (0.46, 0.59)	0.62 (0.55, 0.75)	15.23	<0.001	NC, GP 1 <GP 2
L-MI/Cr	0.71 (0.53, 0.92)	0.90 (0.74, 0.97)	0.75 (0.58, 0.96)	4.17	0.12	—
R-NAA/Cr	1.64 (1.45, 1.87)	1.48 (1.22, 2.01)	1.49 (1.28, 1.69)	7.20	0.027	NC >GP 2
R-Cho/Cr	0.90 (0.77, 1.08)	0.85 (0.67, 1.14)	0.91 (0.80, 1.08)	1.20	0.55	—
R-NAA/Cho	1.87 (1.49, 2.24)	1.74 (1.57, 1.87)	1.54 (1.36, 1.85)	10.92	0.004	NC >GP 2
R-Cho/NAA	0.54 (0.45, 0.67)	0.58 (0.54, 0.64)	0.65 (0.53, 0.74)	9.98	0.007	NC <GP 2
R-MI/Cr	0.70 (0.35, 1.00)	0.90 (0.46, 1.16)	0.75 (0.50, 1.07)	1.35	0.51	—

### Associations between metabolite concentrations and neuropsychological measures

To explore the relationships between the clinical characteristics and ^1^H-MRS data in GP group, we analyzed the possible correlation of the metabolite concentrations with MMSE. There was no correlation between metabolite ratios and MMSE scores in hippocampus regions (Table 5).

**TABLE 5:** Associations between metabolite concentrations and the neuropsychological measures in GP group.

		MMSE	
	r	*p*	Corrected p
L-NAA/Cr	0.282	0.011	0.08
L-Cho/Cr	−0.117	0.30	0.33
L-NAA/Cho	0.224	0.046	0.09
L-Cho/NAA	−0.224	0.046	0.09
L-MI/Cr	−0.136	0.23	0.31
R-NAA/Cr	0.131	0.25	0.31
R-Cho/Cr	−0.130	0.25	0.31
R-NAA/Cho	0.272	0.015	0.08
R-Cho/NAA	−0.254	0.023	0.08
R-MI/Cr	−0.066	0.56	0.56

## Discussion

Neurosyphilis traditionally encompasses five main groups: asymptomatic neurosyphilis, syphilitic meningitis, meningovascular syphilis, general paresis, and tabes dorsalis. General paresis, the chronic encephalitic form of neurosyphilis (Ha et al., 2024). GP is a noteworthy cause of dementia, and while diagnosis has historically relied on clinical manifestations and laboratory findings, neuroimaging and neuropsychological tests can offer diagnostic insights and aid in understanding the disease’s course and prognosis (Wang et al., 2011; Ha et al., 2024). Cortical atrophy, a common finding in neurosyphilis, was also observed in our study. Most GP patients exhibited varying degrees of diffuse atrophy, as evidenced by ^1^H-MRS and MRI results. Cerebral atrophy, likely a consequence of neuron loss, contributes to dementia (Chiara et al., 2023).

Our findings revealed men account for a larger proportion of those with GP. Syphilis spirochetes can directly cause neurological damage, and the activation of the aberrant immune system induced by these spirochetes is believed to indirectly harm target tissues and organs (Carlson et al., 2011). Retrospective searches identified sex as a risk factor for neurosyphilis, with men being more prone to developing neurosyphilis among untreated syphilis cases in clinics. The gender gap may stem from immune system disparities, though other factors such as the influence of traditional Chinese culture, where women tend to be more conservative, reducing syphilis infection rates, and differences in the frequency of testing and treating the infection also play a role (Ta and Se, 2019).

### Patterns of regional metabolite change in patients with GP

^1^H-MRS stands out among imaging modalities as it provides both qualitative and quantitative insights into the biochemical composition of brain tissue. This technique is valuable for assessing neuronal integrity, treatment effects, and exploring new therapies (Graff-Radford et al., 2014).

NAA is regarded as a marker of neuronal integrity (Tomiyasu and Harada, 2022). In our study, significantly lower NAA/Cr ratios were observed in the bilateral hippocampus region of subjects with GP, reflecting neuronal loss or compromised neuronal metabolism, particularly evident in patients with moderate to severe dementia.

Cr is linked to high-energy phosphate metabolism, and its levels tend to rise when energy metabolism declines. While certain research indicates that elevated Cr levels may signify a compensatory response to NAA loss, other studies utilize Cr as a reference due to its relatively stable concentration (Zeng et al., 2021; Tomiyasu and Harada, 2022).

Both hemispheres of the hippocampus in the GP group exhibited lower NAA/Cr ratios compared to the NC group, with a more pronounced trend noted in the left hippocampus region. Particularly noteworthy was the significant difference in NAA/Cr ratios observed among various severity groups within the GP cohort. Numerous studies support the role of NAA/Cr as a marker of neural functional integrity, suggesting that a decreased NAA/Cr ratio in the hippocampus indicates a greater loss of nerve cells. The observed pathophysiological mechanisms are attributed to the proliferation of *T. pallidum*, resulting in the occlusion of small blood vessels, hypoxic conditions, collapse of vessel walls, and reduced cerebral blood flow in the temporal lobe and limbic system (Czarnowska-Cubała et al., 2013; Xiang et al., 2013; Shang et al., 2020).

In our study, the more significant decrease in NAA/Cr ratios observed in patients with moderate to severe dementia suggests pronounced neuronal damage, which may be less apparent in the early stages of dementia. Results for individuals within the normal to mild general paresis range are inconclusive. This variability could be attributed to the differing extents of affected brain regions at various stages of dementia. Dementia associated with neurosyphilis is characterized by cortical hypointensity throughout the entire cortical thickness and brain atrophy, primarily impacting the frontal and temporal lobes. Patients with neurosyphilis-related dementia commonly exhibit clinically significant neuropsychiatric disturbances, including anger, aggression, irritability, and apathy, which are indicative of frontal lobe lesions (Zhong et al., 2017; Ha et al., 2024). As the disease progresses, spirochetal deposition may increase and gradually spread to other brain regions, causing extensive lesions in the temporal lobe or diffuse brain atrophy. And then, it shows significant abnormalities in brain metabolism and cognitive impairment. Thus, GP display a distinct metabolic pattern with a significant decrease in NAA and neuron count as the disease progresses. ^1^H-MRS emerges as a valuable tool for monitoring clinical progression in patients.

Choline is a rate-limiting precursor in acetylcholine synthesis and a component of cell membrane phosphatidylcholine, often used as a marker for cellular density and membrane turnover (Maul et al., 2020; Tomiyasu and Harada, 2022). Moreover, the higher choline content in astrocytes and oligodendrocytes compared to neurons may suggest alterations in membrane phospholipids in GP, contributing to abnormal membrane repair processes, synaptic loss, and the formation of amyloid beta peptide. Higher choline contents have been detected in microglia-rich brain regions such as the thalamus (Urenjak et al., 1993; Plummer et al., 2023). The observed reduction in NAA/Cho and NAA/Cr in our patients reflects changes in membrane metabolism and neuronal loss.

Myoinositol is considered a glial marker, and elevated MI levels can result from increased glial content or activation (Tomiyasu and Harada, 2022). However, in our study, there was no significant difference in the MI/Cr ratios between the GP and NC groups in the bilateral hippocampus region. This suggests that gliosis in the bilateral hippocampus among GP patients may not be as severe as in AD patients, leading to nonsignificant differences in MI/Cr ratios among groups.

The lack of significant differences in Cho/Cr ratios among groups may be attributed to various factors. Cho/Cr ratios have shown inconsistent results in different studies. Different diseases, Brain regions, disease severity and course were influence factors (Wang et al., 2015; Joyce et al., 2022; Guan et al., 2022). In the future, other regions of the brain could be included to assess the significance of Cho/Cr ratios.

### The relationships between neuropsychological measures and the metabolite concentrations in GP

Our study revealed significantly lower neuropsychological assessment scores (MMSE) in the GP group compared to the NC group. However, within the GP group, no correlation was observed between metabolite ratios and MMSE scores in hippocampal regions. This does not imply that ^1^H-MRS may not reflect the cognitive function of GP patients. One possible explanation is that a subset of patients with general paresis are more prone to experiencing diffuse cerebral atrophy. Moreover, syphilis can lead to cerebral vascular syndrome (Ha et al., 2024). It may have characteristics of vascular cognitive impairment such as white matter lesions, unlike AD, is not primarily characterised by medial temporal lobe or hippocampal atrophy. And some GP patients present with mental symptoms that are manifestations of frontal lobe syndrome. Another reason is that MMSE test is be preliminary screening scale for cognitive impairment, encompassing domains such as memory, attention, language ability, calculation and spatial ability. This test requires short time relatively, which is beneficial for patients to cooperate. However, MMSE test evaluates cognitive domains are limited which absences of testing for executive function. MMSE is lack of sensitivity for screening the early stage of dementia (Hammant et al., 2023). Thus, there was no correlation between MMSE scores and metabolite ratios.

Researchers have shown distinct patterns of CSF biomarkers (Aβ and tau) in GP and AD patients. The unique CSF Aβ pattern in general paresis suggests abnormal Aβ metabolism (Luo et al., 2015; Chiara et al., 2023). This raises questions about the potential association of GP pathogenesis with neurodegenerative factors. In clinical practice, a subset of GP patients does not experience improved cognitive function after penicillin antisyphilitic treatment. This prompts inquiries into whether these patients may have concurrent AD. Exploring the use of drugs employed for AD treatment, such as cholinesterase inhibitors, in GP patients warrants consideration. Our study identified a higher proportion of male patients in the GP group than females, prompting speculation about potential protective factors in females against syphilis. The exploration of metabolic characteristics in multiple brain regions in GP could enhance our understanding and guide treatment strategies. In conclusion, further studies are crucial to comprehensively understand the extent and persistence of brain injuries in GP.

There were several limitations that warrant consideration. The brain regions associated with cognitive functions include the frontal lobe, temporal lobe, parietal lobe, corpus callosum, among others. However, the clinical manifestations of patients with GP are diverse. In this study, we solely analyzed the levels of metabolites in the hippocampus. It is worth noting that different regions of brain damage may exhibit distinct metabolic characteristics. Future research could investigate the metabolic profiles of multiple brain regions and the concentrations of individual metabolites in patients with GP to obtain a more comprehensive understanding of the metabolic alterations in the GP brain.

## Conclusion

Hydrogen proton magnetic resonance spectroscopy is a non-invasive method used to assess neuronal function in GP. GP patients exhibited significantly lower NAA/Cr and NAA/CHO ratios in the hippocampus, particularly in moderate and severe dementia period, indicating neuron loss in these areas, which may become more pronounced as the disease progresses, suggesting distinctive metabolic characteristics in the hippocampus of GP patients. Our findings highlight neuronal injuries in the bilateral hippocampus of GP patients. Research on the metabolic characteristics of multiple brain regions is essential for better-informed treatment approaches. Our ongoing goal is to enhance treatment strategies for GP through continued research.

## Data Availability

The raw data supporting the conclusion of this article will be made available by the authors, without undue reservation.
